# Assessment of trends and determinants of under-five mortality among children born to older women: Evidence from Ethiopian demographic and health surveys

**DOI:** 10.1371/journal.pone.0319447

**Published:** 2026-07-23

**Authors:** Tamerat Denekew Temesegen, Tariku Dejene Demissie, Solomon Abrha Damtew

**Affiliations:** 1 Tamerat Denekew, Health and Social Research Consultancy, Addis Ababa, Ethiopia; 2 Center for Population and Gender Studies, College of Social Sciences, Arts, and Humanities, Addis Ababa University, Addis Ababa, Ethiopia; 3 Department of Epidemiology and Biostatistics, School of Public Health, St Paul’s Hospital Millenium Medical College, Ministry of Health, Ethiopia; Chittagong Medical College, BANGLADESH

## Abstract

**Introduction:**

Under-five mortality has long been used as an indicator of the level of socio-economic development of a country. Global attention has been given to the issue through the Sustainable Development Goals, yet the burden continues to be disproportionately concentrated in Sub-Saharan Africa and South Asia. Ethiopia is one of six nations that together account for half of global under-five mortality, with particular burden observed among children born to older mothers. Hence, this study aimed to determine the magnitude of under-five mortality among children born from elder mothers, assess its trend over time, and identify associated factors.

**Methods:**

Data were drawn from the nationally representative Ethiopia Demographic and Health Surveys (EDHS) conducted in 2000, 2005, 2011, and 2016. The analytic sample comprised 6,199 children born to women aged 35–49 years in the five years preceding each survey. Information on mother and child characteristics, socio-economic factors, and environmental variables was collected through face-to-face household interviews. Descriptive statistics characterized the study participants. Multilevel binary logistic regression was used to estimate adjusted odds ratios and 95% confidence intervals for factors associated with under-five mortality.

**Result:**

Under five mortality declined from 128 per 1000 live births during the year 2000–56 per 1000 live births in 2016. This study identified several factors contributing for under-five mortality among children of women aged 35–49 years. Protective factors included female child sex (AOR = 0.65, 95% CI: 0.53–0.79), maternal age at first birth of 31–40 years (AOR = 0.34, 95% CI: 0.11–0.98), family size of 6–10 members (AOR = 0.18, 95% CI: 0.14–0.24), birth interval greater than 3 years (AOR = 0.35, 95% CI: 0.26–0.47), and 1–3 antenatal care visits (AOR = 0.65, 95% CI: 0.50–0.84). In contrast, the following factors increased the odds: twin birth (AOR = 6.15, 95% CI: 3.94–9.60), four births in the last five years (AOR = 2.64, 95% CI: 1.15–6.06), nine or more children ever born (AOR = 3.79, 95% CI: 2.10–6.84), and maternal age at birth of the index child of 45–49 years (AOR = 2.13, 95% CI: 1.55–2.93).

**Conclusion:**

The decline in under-five mortality over the last 16 years in Ethiopia represents promising progress toward the targets set out in the Sustainable Development Goals and national health policy. However, targeted programs and interventions focusing on provision of the recommended antenatal care, adequate birth spacing, limiting the number of births within short intervals, discouraging pregnancies after age 40, and supporting smaller family sizes are essential to further reduce under-five mortality. The study findings highlight the importance of risk-pregnancy screening, well-spaced pregnancies, family health promotion, and strengthened childhood illness prevention and treatment services.

## Background

The World Health Organization defines under five mortality as the death of children born during a specific year or period before reaching the age of 5 years [1]. Under five mortality serves as a critical indicator of the country’s heath system infrastructure, health equity, and broader challenges in economic growth and development, building on the successes of Millennium Development Goals (MDGs) [2,3]. Sustainable Development Goal (SDG) 3 target 3.2 aims to end preventable deaths of newborns and children under 5 years of age, with all countries striving to reduce under-5 mortality to at least 25 deaths per 1,000 live births by 2030 [4,5].

Ethiopia is one of the East African countries that demonstrated remarkable progress in achieving the MDGs, particularly through a substantial reduction in under-five mortality. The national under-five mortality rate stood at 67 deaths per 1000 live births, according to the Ethiopian Demographic and Health Survey 2016. Under-five mortality in the country fell from 166 deaths per 1000 live births in 2000–67 deaths per 1000 live births in 2016, representing a 60% decline over 16 years [6]. More recent evidences show that the rate has further decreased to 51 deaths per 1,000 live births [7–10]. Nevertheless, the pace of decline has not been as rapid as hoped, and the rate remains unacceptably high, making under-five mortality one of the most pressing public health challenges facing the country.

In response to this challenge, the Federal democratic republic of Ethiopia Health Mister have designed and implementing various policies and strategies to reduce under five mortality as clearly speculated in the country´s Newborn care guidelines and in the integrated management of Common childhood illness guideline [11–13]. These commitments area also reflected in the country´s recent health sector transformation plan. Key initiatives include improvements in preventive and curative service provision, notably free of charge under five children treatment at health post level as part of the primary health care unit [14].

Additionally, expanded access to modern health services for the mother during pregnancy, childbirth, and postnatal period has contributed to the reduction in under-five mortality. The government has demonstrated further commitment for newborn care by establishing newborn care units (NICUs) and teaching hospitals train midlevel professionals such as neonatal nurses and resident programs fill in the gap in skilled man power [15,16]. The introduction of health insurance scheme is another national commitment to health care equity and universal access [17–19].

While several studies have examined the factors and magnitude of under-five mortality [20–23], none have specifically quantified under-five mortality among children born to mothers aged 35–49 years. Among these group, the burden is particularly pronounced and identifying the factors that contribute to under-five mortality among older women is essential for targeting interventions to the most vulnerable population segments and accelerating further reductions in death rates. Although progress has been made, additional efforts are required to overcome remaining barriers to child survival. Therefore, this study aimed at determining the magnitude of under-five mortality among children born from elder mothers among whom the death is pronounced; and identifying the associated correlates and assess its overall trend using a four data point from the Ethiopian Demographic and Health Survey (EDHS). The findings are expected to provide actionable evidence to support the Federal Ministry of Health and its partners in tracking progress toward the achievement of Sustainable Development Goal (SDG) 3.2, strengthening child survival programs, and contributing to reductions in both under-five mortality and maternal mortality while improving newborn health [5].

## Methods

This study used data from four consecutive Ethiopia Demographic and Health Surveys (EDHS) conducted in 2000, 2005, 2011, and 2016. The surveys were implemented by the Central Statistical Agency (CSA) of Ethiopia in collaboration with the Federal Ministry of Health (FMoH) and the Ethiopian Public Health Institute (EPHI), with technical assistance from ICF International and financial/technical support from development partners. They were designed to provide nationally representative estimates of key health and demographic indicators for Ethiopia as a whole, for urban and rural areas separately, as well as for the 11 administrative domains [8–10]. The samples comprised 2,124 births from the 2000 survey, 1,746 from the 2005 survey, 1,657 from the 2011 survey, and 1,632 from the 2016 survey. These were extracted from the birth histories of 35–49 years old mothers for the five years preceding the survey.

The outcome variable was under-five mortality among children born to older women, defined as death before the fifth birthday. This binary outcome was coded as 1 if the child died before age 5 and 0 if the child survived. The explanatory variables were selected from literature on similar studies on child morbidity and mortality in developing countries, and data availability. These variables were grouped into demographic, socioeconomic, and environmental domains.

The demographic factors included sex of child (1 = male, 2 = female), age of child in years, type of birth (0 = single, 1 = multiple), birth order (1 = first, 2 = 2–4, 3 = ≥5), preceding birth interval in months (0 = first birth, 1 = < 24, 2 = 24–35, 3 = ≥36), mother’s age at first birth in years (1 ≤ 20, 2 = 21 30, 3 =  31 32 –39 40, 4  ≥ 41), family size (1 =  1 2 –45, 2  =  6 7 –910, 3  ≥ 11), and breastfeeding status (1 = ever breastfed, 2 = never breastfed). The socioeconomic factors comprised mother’s education level (0 = no education, 1 = primary, 2 = secondary or higher), wealth index (1 = poor, 2 = middle, 3 = rich), administrative region (1 = Tigray, 2 = Afar, 3 = Amhara, 4 = Oromia, 5 = Somali, 6 = Benishangul-Gumuz, 7 = SNNPR, 8 = Gambela, 9 = Harari, 10 = Addis Ababa, 11 = Dire Dawa), and place of residence (1 = urban, 2 = rural). The list of environmental factors included toilet facility (0 = improved, 1 = unimproved, 2 = none), source of drinking water (0 = protected, 1 = unprotected), and place of delivery (0 = home, 1 = health facility).

After data were checked for completeness, inconsistencies, missing values and outliers the analysis were performed using STATA version 16.0 statistical software. All estimates were weighted to account for the complex survey design and unequal selection probabilities. Frequencies and proportions were computed for description of the study population in relation to selected socio-demographic variables. The results were presented in the form of tables and figures.

Multilevel binary logistic regression was used to analyze the data. At bivariate level, variables with a p-value ≤ 0.25 were retained as candidate variable for multilevel multivariable logistic regression analysis. Results were presented in the form of odds ratio with 95% CI. Statistical significance was declared at a p-value less than 0.05. Four models were run; the first was the intercept only model in which no factors were included following which intra-cluster correlation coefficient (ICC) was computed to check the level of clustering of observations among enumeration areas (EAs). The clustering was found to be 28.8% which was far beyond the conventional cut off point, 0.05 for the fulfillment of independent observation assumption; hence, the use of multilevel logistic regression over the single level modeling was justified [24–26]. In the second model, individual level variables were introduced while only enumeration area level variables were entered in the third model. In the final model, both individual and enumeration area level independent variables were modeled. For each model, ICC, Akaike and Bayesian information criteria (AIC and BIC) along with log likelihood values were computed. Based on the result, the final model with lower AIC and BIC along with higher likelihood was selected as best fitted model from which the adjusted odds ratio were computed and reported.

This study was based on secondary data obtained from the Demographic and Health Surveys (DHS) Program. The procedures and questionnaires for the standard DHS were reviewed and approved by the ICF/ORC Institutional Review Board. In addition, the survey protocols received ethical clearance from the Institutional Review Board of the Ministry of Science and Technology of Ethiopia and the Ethiopian Health and Nutrition Research Institute. All interviews were conducted only after obtaining oral informed consent from respondents. To safeguard participant anonymity, names and all individual identifiers were removed from the final dataset.

## Result

Table 1 presents the distribution of births occurring in the five years preceding each survey among women aged 35–49 years by selected child, maternal, and household characteristics. The proportion of births to mothers whose first birth occurred at or before age 20 years declined from 69.4% in 2000 to 61.2% in 2016, while the proportion whose first birth occurred between ages 21 and 30 years rose from 29.2% to 35.3%. In regards to birth order, 88.0–93.8% of index children were of fifth or higher order across surveys, although the share of lower-order births [3,4] rose modestly over time. Long preceding birth interval births (≥37 months) were predominant and increased slightly from 54.6% in 2000 to 58.5% in 2016. Sex of the index child was roughly balanced across years, and twin births remained rare (2.3–3.9%).

**Table 1:** Distribution of births to older women by basic characteristics and survey year, Ethiopia.

Variables and categories	Survey Year			
	2000 N (%)	2005 N (%)	2011 N (%)	2016 N (%)
**Total**	**2124**	**1746**	**1657**	**1632**
**Age of the mother at first birth**				
<=20	1,474 (69.4)	1,098 (62.9)	1,042 (62.9)	998 (61.2)
21-30	621 (29.2)	613 (35.1)	571 (34.5)	575 (35.3)
31-40	28 (1.3)	34 (1.8)	44 (2.7)	58 (3.6)
**Sex of index child**				
Male	1,137 (53.5)	842 (48.2)	875 (52.8)	813 (49.8)
Female	987 (46.5)	905 (51.8)	781 (47.2)	819 (50.2)
**Type of birth**				
Single	2,072 (97.6)	1,679 (96.2)	1,619 (97.7)	1,568 (96.1)
Twin	52 (2.4)	67 (3.8)	38 (2.3)	63 (3.9)
**Birth order of index child**				
1st birth	6 (0.3)	6 (0.3)	15 (0.9)	8 (0.5)
2nd birth	12 (0.6)	17 (0.9)	32 (1.9)	25 (1.5)
3-4 births	115 (5.4)	103 (5.9)	99 (6.0)	163 (10.0)
5 and above Births	1,991 (93.8)	1,621 (92.8)	1,511 (91.2)	1,436 (88.0)
**Preceding birth interval**				
Up to 23 Months	274 (12.9)	225 (13.0	212 (12.9)	254 (15.6)
24-36 Months	689 (32.5)	504 (29.0)	471 (28.7)	421 (25.9)
More than 37 Months	1,156 (54.6)	1,011 (58.1)	958 (58.4)	949 (58.5)
**Highest education level of the mother**				
No education	2,017 (95.0)	1,580 (90.5)	1,315 (79.4)	1,346 (82.5)
Primary	82 (3.9)	132 (7.6)	320 (19.3)	240 (14.5)
Secondary	21 (1.0)	28 (1.6)	13 (0.8)	27 (1.7)
Higher	4 (0.2)	6 (0.3)	9 (0.5)	17 (1.04)
**Place of residence**				
Urban	145 (6.8)	88 (5.0)	175 (10.6)	128 (7.9)
Rural	1,979 (93.2)	1,658 (95.0)	1,482 (89.4)	1,503 (92.1)
**Source of drinking water**				
Piped	190 (8.9)	264 (15.1)	399 (24.1)	376 (23.0)
Tube Well	1211 (57.0)	198 (11.3)	68 (4.1)	214 (13.1)
Dug	678 (31.9)	715 (41)	203 (12.2)	204 (12.5)
Spring	–	554 (31.7)	926 (55.9)	785 (48.1)
Other	44 (2.1)	14 (0.8)	62 (3.7)	53 (3.2)
**Type of toilet facility**				
No Facility	1885 (88.8)	1211 (69.3)	686 (41.4)	637 (39.0)
Flush	3 (0.2)	16 (0.1)	27 (1.7)	28 (1.7)
Pit latrine	191 (9.0)	511 (29.4)	840 (50.7)	930 (57)
Other	44 (2.1)	9 (0.5)	104 (6.3)	36 (2.2)
**Place of delivery**				
Health Facility	71 (3.4)	61 (3.5)	107 (6.4)	347 (21.2)
Home	2053 (96.7)	1686 (96.5)	1550 (93.6)	1285 (78.7)
**Number of antenatal visit**				
No Antenatal Visit	1289 (78.1)	1065 (76.6)	823 (62.9)	624 (47.0)
Up To 4 Visit	267 (16.2)	241 (17.3)	384 (29.4)	527 (39.8)
More than 4 Visit	93 (5.7)	85 (6.1)	101 (7.7)	175 (13.2)
**FP current use**				
No Method	1982 (93.3)	1550 (88.8)	1358 (81.8)	1180 (72.3)
Use FP Method	142 (6.7)	196 (11.2)	299 (18.0)	452 (27.7)

Births to mothers with no formal education decreased from 95.0% in 2000 to 82.5% in 2016, while the share of births to mothers with primary education rose from 3.9% to 14.5%. The proportion of births to mothers using family planning quadrupled from 6.7% in 2000 to 27.7% in 2016. The proportion of births with no antenatal visits fell from 78.1% to 47.0%, and the corresponding share for antenatal care of more than four visits had more than doubled (5.7% to 13.2%). Institutional delivery rose from 3.4% in 2000 to 21.2% in 2016. The large majority of births (89.4–95.0%) occurred in rural households. Similarly, the proportion of births happening in households with piped water increased from 8.9% to 23.0% and the proportion of births in households using spring as a water source has become significantly higher (48.1–55.9%) in later surveys. The births in households with no toilet facility declined sharply from 88.8% in 2000 to 39.0% in 2016, while the births in households with pit-latrine rose from 9.0% to 57.0% (Table 1).

**Fig 1:**
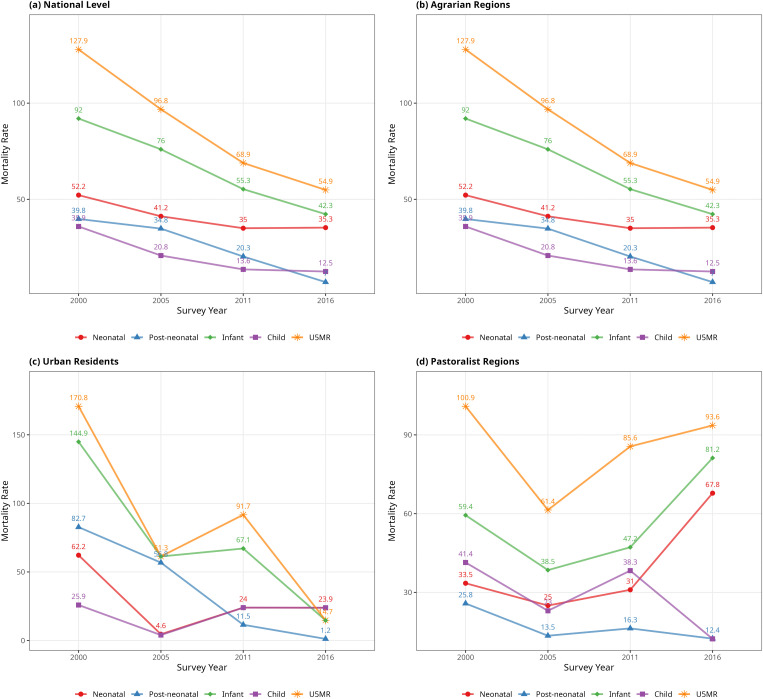
Trend of under-five, neonatal, post neonatal, infant and child mortality among children born to older women.

At the national level, under-five mortality (U5MR) among children of older women declined steadily and substantially from 127.9 deaths per 1,000 live births in 2000 to 54.9 in 2016, representing a reduction of more than 57%. Infant mortality followed a similar downward trajectory, falling from 92 to 42.3 per 1,000 live births. Neonatal mortality decreased from 52.2 to 35.3 per 1,000, although the decline slowed after 2011 and the rate plateaued. The most striking improvement occurred in post-neonatal mortality, which dropped sharply from 39.8 to 7.0 per 1,000 live births. Child mortality (ages 1–4 years) also fell markedly from 35.9 to 12.5 per 1,000, with the largest gains occurring between 2000 and 2011. Overall, the national trends demonstrate consistent and substantial progress across all mortality indicators, with the greatest absolute reductions observed in the post-neonatal and child mortality components (Fig 1(a)).

Trends in mainly agrarian regions closely mirrored the national pattern. U5MR declined from 127.9 per 1,000 live births in 2000 to 54.9 in 2016, while infant mortality decreased from 92 to 42.3 per 1,000. Neonatal mortality fell from 52.2 to 35.3 per 1,000, stabilizing after 2011. Post-neonatal mortality showed the most dramatic improvement, plummeting from 39.8 to 7.0 per 1,000 live births. Child mortality declined from 35.9 to 12.5 per 1,000. These parallel declines indicate that the majority of the national progress was driven by improvements in the agrarian regions, which constitute the bulk of Ethiopia’s population (Fig 1(b)).

Urban areas started with markedly higher mortality rates in 2000 but exhibited the most rapid overall improvement by 2016. U5MR declined sharply from 170.8 per 1,000 live births in 2000 to 14.7 in 2016. Infant mortality fell from 144.9 to 23.9 per 1,000, while post-neonatal mortality dropped dramatically from 82.7 to 1.2 per 1,000. Neonatal mortality decreased from 62.2 to 23.9 per 1,000, although the decline was interrupted by a temporary rise in 2011. Child mortality fell from 25.9 to 23.9 per 1,000. Despite some data gaps in 2005, the urban trends reveal exceptionally steep reductions after 2011, resulting in the lowest absolute mortality rates observed across all domains by the end of the study period (Fig 1(c)).

In contrast to the other domains, mortality trends in mainly pastoralist regions were more volatile and showed less overall improvement. U5MR declined from 100.9 per 1,000 live births in 2000 to 61.4 in 2005, but then rose again to 93.6 by 2016. Infant mortality followed a similar pattern, falling from 59.4 to 38.5 before increasing to 81.2 per 1,000. Neonatal mortality decreased initially (33.5 to 25.0) but then surged to 67.8 per 1,000 in 2016. Post-neonatal mortality remained relatively low and stable (25.8 to 12.4 per 1,000). Child mortality showed an initial decline followed by fluctuation, ending at 12.4 per 1,000 in 2016. These patterns indicate persistent or worsening vulnerabilities in pastoralist regions, particularly for neonatal and infant mortality, despite modest gains in the early 2000s (Fig 1(d)).

Table 2 presents the adjusted odds ratios (AOR) and 95% confidence intervals from the final multilevel multivariable logistic regression model (Model III), which simultaneously adjusted for individual-level and enumeration-area-level factors while accounting for clustering of children within enumeration areas. Female children had 35% lower odds of under-five mortality compared with male children (AOR = 0.65, 95% CI: 0.53–0.79). Twin births were associated with more than six-fold higher odds of death before age five than singleton births (AOR = 6.41, 95% CI: 4.10–10.02). Compared with mothers who had only one birth in the five years preceding the survey, the odds of under-five mortality were progressively and significantly elevated among those with two births (AOR = 1.33, 95% CI: 1.05–1.70), three births (AOR = 1.53, 95% CI: 1.00–2.33), and four births (AOR = 2.56, 95% CI: 1.11–5.90).

**Table 2:** Multivariable multilevel logistic regression on factors contributing to under-five mortality among children born to women aged 35 to 49 in Ethiopia.

Variables	Category	Alive	Dead	COR 95% CI	Model l AOR 95% CI	Model ll AOR 95% CI	Model lII AOR 95% CI
**Sex of Child**	Male	3286	381	1	1		1
	Female	3228	264	0.71 (0.59, 0.85)***	0.65 (0.53, 0.79) ***		0.65 (0.53, 0.79) ***
**Birth Type**	Single	6364	575	1	1		1
	Twin	151	69	5.67 (3.88, 8.26) ***	6.43 (4.11, 10.04) ***		6.41 (4.1, 10.02) ***
**Marital Status**	Currently in union	6122	578	1	1		1
	Not in union	392	67	1.9 (1.38, 2.62) ***	1.34 (0.86, 2.11)		1.34 (0.85, 2.1)
**Births in the last 5 Years**	1	3182	3429	1	1		1
	2	2824	297	1.39 (1.14, 1.69) ***	1.33 (1.05, 1.7) *		1.33 (1.05, 1.7) *
	3	462	80	2.24 (1.62, 3.08)***	1.53 (1, 2.33) *		1.53 (1, 2.33) *
	4	46	19	4.61 (2.26, 9.39)***	2.54 (1.1, 5.86) *		2.56 (1.11, 5.9) *
**Age at 1st Birth**	<20	4183	429	1	1		1
	21-30	2174	205	0.87 (0.71, 1.06)	0.83 (0.65, 1.06)		0.83 (0.65, 1.06)
	31-40	156	10	0.73 (0.36, 1.51)	0.36 (0.12, 1.04)		0.35 (0.12, 1.03) *
**Sex of HH head**	Male	5569	523	1	1		1
	Female	945	121	1.48 (1.17, 1.89) ***	0.94 (0.67, 1.31)		0.94 (0.68, 1.32)
**Total Children Ever born**	up to 4 child	518	27	1	1		1
	5 up to 8 Child	3561	291	1.43 (0.93, 2.21)	1.98 (1.16, 3.39) *		1.99 (1.16, 3.42) **
	9 and above child	2434	326	2.53 (1.64, 3.91) ***	3.53 (1.97, 6.35) ***		3.56 (1.97, 6.43) ***
**Family Size**	Up to 5	839	188	1	1		1
	2. 6-10	5155	438	0.33 (0.26, 0.41)***	0.2 (0.15, 0.26) ***		0.2 (0.15, 0.26) ***
	3. >=11	520	18	0.11 (0.06, 0.19)***	0.04 (0.02, 0.07) ***		0.04 (0.02, 0.07) ***
**Preceding Birth Interval**	<24 Months	808	156	1	1		1
	24 - 36 Months	2074	221	0.56 (0.44, 0.72) ***	0.59 (0.45, 0.78) ***		0.59 (0.45, 0.78) ***
	>=37	3601	262	0.35 (0.28, 0.45) ***	0.34 (0.26, 0.46) ***		0.34 (0.26, 0.46) ***
**Age at birth of index** **Child**	35-39	2437	164	1	1		1
	40-44	2954	332	1.81 (1.46, 2.25) ***	1.65 (1.29, 2.1) ***		1.65 (1.3, 2.1) ***
	45-49	1122	149	2.08 (1.6, 2.69) ***	2.09 (1.52, 2.88) ***		2.09 (1.52, 2.88) ***
**Mother’s Education** **Level**	No Education	5681	577	1	1		1
	Primary	718	57	0.81 (0.59, 1.11)	1.16 (0.81, 1.66)		1.15 (0.8, 1.66)
	Secondary and above	115	11	1.03 (0.51, 2.08)	1.32 (0.53, 3.3)		1.28 (0.49, 3.36)
**Total ANC visit**	No ANC visit	3504	297	1	1		1
	Up To 4 Visit	1665	131	0.75 (0.57, 0.99) *	0.73 (0.56, 0.95) *		0.73 (0.56, 0.95) *
	More than 4 Visit	467	44	0.96 (0.62, 1.48)	1.46 (0.96, 2.23)		1.45 (0.94, 2.22)
**Survey Year**	2000	1852	271	1	1		1
	2005	1582	165	0.7 (0.54, 0.9) **	0.76 (0.57, 1)		0.76 (0.57, 1) *
	2011	1541	116	0.55 (0.42, 0.73) ***	0.6 (0.44, 0.82) *		0.6 (0.44, 0.82) **
	2016	1540	92	0.39 (0.29, 0.53) ***	0.46 (0.33, 0.65) *		0.47 (0.33, 0.65) ***
**Region**	Urban	91	8	1		1	1
	Highland	6104	607	1.17 (0.53, 2.56)		1.12 (0.48, 2.61)	0.83 (0.32, 2.11)
	Lowland	319	28	1.1 (0.45, 2.67)		1.06 (0.42, 2.69)	0.79 (0.28, 2.21)
**Place of Residence**	Urban	491	45	1		1	1
	Rural	6023	599	1.08 (0.73, 1.59)		1.05 (0.69, 1.6)	1.02 (0.62, 1.66)

*=p value<0.05 **=p value<0.01 ***= p value<0.001.

Mothers whose first birth occurred at age 31–40 years had 65% lower odds of under-five mortality than those whose first birth was before age 20 (AOR = 0.35, 95% CI: 0.12–1.03). Higher lifetime parity was strongly associated with increased risk: mothers with 5–8 children ever born had nearly twice the odds (AOR = 1.99, 95% CI: 1.16–3.42), and those with 9 or more children had 3.56 times the odds (AOR = 3.56, 95% CI: 1.97–6.43) compared with mothers with four or fewer children ever born (Table 2).

Larger household size was strongly protective. Births in households with 6–10 members had 80% lower odds (AOR = 0.20, 95% CI: 0.15–0.26), and those in households with 11 or more members had 96% lower odds (AOR = 0.04, 95% CI: 0.02–0.07) of under-five mortality relative to households with five or fewer members. Longer preceding birth intervals were also protective: intervals of 24–36 months (AOR = 0.59, 95% CI: 0.45–0.78) and ≥37 months (AOR = 0.34, 95% CI: 0.26–0.46) were associated with substantially lower odds compared with intervals shorter than 24 months (Table 2).

Maternal age at the birth of the index child showed a clear gradient. Children born to mothers aged 40–44 years had 65% higher odds (AOR = 1.65, 95% CI: 1.30–2.10), and those born to mothers aged 45–49 years had more than double the odds (AOR = 2.09, 95% CI: 1.52–2.88) of under-five mortality compared with children born to mothers aged 35–39 years. Regarding health-service utilization, mothers who reported up to four antenatal care (ANC) visits had 27% lower odds of under-five mortality than those with no ANC visits (AOR = 0.73, 95% CI: 0.56–0.95). A clear temporal decline in under-five mortality was observed across survey years: compared with 2000, the adjusted odds were 24% lower in 2005 (AOR = 0.76, 95% CI: 0.57–1.00), 40% lower in 2011 (AOR = 0.60, 95% CI: 0.44–0.82), and 53% lower in 2016 (AOR = 0.47, 95% CI: 0.33–0.65) (Table 2).

**Table:** 

	Null	Individual	Group	Final
**ICC**	0.2973	0.2886	0.29747	0.2880
**PCV**	Reference	0.0294	−0.0005	0.0314
**AIC**	4100.8520	3621.5720	4106.5770	3627.3680
**BIC**	4114.3160	3796.4050	4106.5770	3822.3740
**Variance**	1.39	1.33	1.39	1.33
**log Likelihood**	−2048.4259	−1784.7859	−2048.2884	−1784.6838

## Discussion

The study sought to estimate under-five mortality levels among children born to older mothers, evaluate temporal trends, and identify key determinants using four rounds of the EDHS data. The findings demonstrate substantial national progress, with an under-five mortality rate among this cohort declining by 57% between 2000 and 2016. Parallel downward trajectories were observed across neonatal, post-neonatal, infant, and child mortality sub-indices. These improvements were largely underpinned by consistent gains in mainly agrarian regions, which mirror the national pattern and represent the majority of the population. Multi-variable analysis further identified key risk and protective factors: female children, longer birth intervals, larger household sizes, and antenatal care utilization were significantly associated with reduced odds of under-five mortality. Conversely, twin births, higher parity, and short birth intervals were identified as primary drivers of increased risk among children born to older women.

The substantial reduction in under-five mortality observed over the last fifteen years is consistent with previous research [27] and reflects the successful realization of the Millennium Development Goals (MDGs) alongside rigorous ongoing efforts to meet the Sustainable Development Goal (SDG) targets [4]. This progress is primarily anchored in the high-level commitment of the Ethiopian government to articulate, implement, and monitor comprehensive health policies and strategies, such as the Essential Newborn Care (ENC) initiatives and the Integrated Management of Childhood Illness (IMCI) frameworks [11,28] as well as the strategic successes achieved under the Health Sector Transformation Plan (HSTP II) [29]. Technically and operationally, several supply-side and community-level interventions have catalyzed this decline, including improved maternal and newborn health service access through the provision of delivery services free of charge [16,29].

Furthermore, the effectiveness of the Health Extension Program has been pivotal in bringing essential care to the community level [14,15], while a significant expansion of Primary Health Care Units (PHCUs) and primary hospitals has strengthened the health infrastructure. This expansion includes building the capacity of existing facilities through the establishment of Neonatal Intensive Care Units (NICU), the expansion of emergency and obstetric surgery programs, and the introduction of specialty nursing programs, such as neonatal nursing [3,14,30]. Additionally, sustained efforts by the Ministry of Health to improve the overall quality of care, coupled with increased access to contraceptive commodities to encourage birth spacing, have further reduced mortality risks [31,32].

The finding that higher birth order significantly increases the odds of under-five mortality is consistent with previous research conducted in various contexts [21,23,33,34]. However, this result diverges from findings in other sub-Saharan African settings; for instance, evidence from Ghana suggested that higher birth order was associated with lower mortality risks [35]. The discrepancy between these findings may be attributed to variations in socioeconomic status and healthcare coverage between Ethiopia and Ghana. Such differences often stem from distinct sociocultural landscapes and varying degrees of male dominance in fertility decision-making, which can influence maternal health-seeking behavior and resource allocation within the household [22]. Consequently, in the Ethiopian context, the increased risk associated with higher birth order underscores the physiological and economic strain placed on both the mother and the family unit, particularly among older women who may already face heightened reproductive risks.

The finding that a higher number of births in the five years preceding the survey increased the odds of under-five mortality in this study stands in contrast to evidence from Ghana, where a higher frequency of recent births was associated with lower mortality odds [35]. Similarly, twin births were found to significantly increase the risk of under-five death, aligning with established research [20,33]. This differs from the Ghanaian context, where multiple births appeared to lower mortality risk [35], although other studies have consistently reported that singleton births carry significantly lower odds of mortality [21,34]. These discrepancies likely stem from variations in study settings and healthcare infrastructure between Ethiopia and Ghana.

Regarding maternal healthcare, this study found that having fewer than four antenatal care (ANC) visits was associated with lower mortality odds. This is a notable departure from several studies [23,36,37] which reported that adhering to the recommended number of visits reduced child mortality. Despite these conflicting results, the protective effect of ANC utilization observed here generally aligns with the World Health Organization’s recommendations on the continuum of care as a driver for better pregnancy and delivery outcomes. Furthermore, wider birth intervals were found to lower the odds of mortality, consistent with existing literature [20,37], though some research has conversely suggested that longer intervals increase risk [20] or that shorter intervals are the primary drivers of mortality [34].

Interestingly, this study revealed that large household sizes were associated with lower odds of under-five mortality. This contradicts traditional findings where larger families increased mortality risk due to resource dilution [20,21,33]. Regarding the sex of the child, being female was found to be a protective factor in this study, which aligns with some research [37] but contrasts with findings in Ghana [35] and other studies [23] where being female or male was either associated with higher risk or found to be statistically insignificant [20,34].

Factors such as breastfeeding, source of drinking water, and maternal income were not found to be significant in this study, diverging from previous evidence [20]. This lack of significance may be due to the specific study population, which was restricted to older women (aged 35–49), whereas other studies typically include the entire reproductive age range. Similarly, while educational status and residence did not significantly influence mortality in this cohort, aligning with some findings [20,23], other studies have reported that rural residence [33,38,39], lack of education [34], and home delivery [21] significantly increase the risk of death. Finally, unlike some literature where contraceptive use, private facility delivery, and marital status were associated with increased mortality [23], these variables did not show similar effects in this specific Ethiopian context.

While this study utilizes nationally representative data to provide a comprehensive overview of mortality trends, it is not without limitations. The secondary nature of the dataset presents inherent drawbacks, as the researchers are constrained by the variables and data collection methods. Furthermore, because the data were collected through a cross-sectional design, the findings may be susceptible to recall and social desirability biases, which are common in retrospective self-reporting. Additionally, the temporal trends were estimated based on four distinct snapshot points rather than longitudinal tracking, which may not capture a perfectly fluid picture of mortality changes over time. However, to mitigate this, the survey year was explicitly integrated into the multivariable modeling process. The significant association found between the survey year and under-five mortality among older women suggests that, despite these snapshot-based limitations, the model effectively captures a statistically robust decline in mortality over the study period. We also note that the most recent data available at the time of analysis were from the 2016 EDHS. This limits insights into trends post-2016, and acknowledge constraints inherent to publicly available survey datasets.

## Conclusions

The study demonstrates that Ethiopia has achieved a monumental 57% reduction in under-five mortality among children born to older mothers between 2000 and 2016. This substantial national progress mirrors improvements across neonatal, post-neonatal, infant, and child mortality components and was particularly pronounced in agrarian regions. Key protective factors identified in the multivariable analysis, female child sex, longer birth intervals, larger household sizes, and antenatal care utilization, have contributed to these gains. Nevertheless, approximately 1 in 19 children in the most recent cohort still die before their fifth birthday. High-risk factors, including twin births, higher parity, short birth intervals, and advanced maternal age at index birth, continue to elevate mortality odds in this vulnerable group.

While these achievements reflect successful implementation of national strategies such as the Health Extension Program, Essential Newborn Care, IMCI, and HSTP II, sustained and more targeted efforts are required to meet SDG 3.2 targets. The Ministry of Health and partners should shift from broad child survival approaches toward high-precision interventions. These include enhanced risk-pregnancy screening for older mothers, promotion of optimal birth spacing and limiting high parity, strengthened capacity at primary hospitals and NICUs for essential newborn care, and integration of community-level predictive screening through the Health Extension Program. Incorporating qualitative insights from frontline implementers and leveraging recent data should be critical. By addressing the specific reproductive health needs of older mothers within a resilient, equity-focused healthcare framework, Ethiopia can build on these promising trends to ensure the survival and thriving of every child, irrespective of maternal age or geographic context.

## Abbreviations

AAU: Addis Ababa University
ANC: Antenatal care
CoDS: College of Developmental Studies
COVID: Coronavirus Disease
CSA: Central Statistical Agency
CM: Child mortality
CPR: Contraceptive Prevalence Rate
DHS: Demographic and Health Survey
EA: Enumeration Area
EDHS: Ethiopian Demographic and Health Survey
EPHI: Ethiopian Pubic Health Institute
EPHC: Ethiopian Population and Housing Census
GTP: Growth Transformation Plan
HBM: Health Belief Model
HH: House Hold
IM: Infant mortality
MDG: Millennium Development Goal
MoH: Ministry of Health
NGO: Non-Governmental Organization
NICU: Neonatal Intensive Care Unit
NM: Neonatal mortality
NMR: Neonatal mortality Rate
PNM: Post Neonatal mortality
SDG: Sustainable Development Goal
SSA: Sub-Saharan Africa
SNNPR: Southern Nation and Nationalities Region
UN: United Nation
UNICEF: United Nations International Children’s Emergency Fund
U5M: Under-five mortality
WHO: World Health Organization
